# Standardized procedure to measure the size distribution of extracellular vesicles together with other particles in biofluids with microfluidic resistive pulse sensing

**DOI:** 10.1371/journal.pone.0249603

**Published:** 2021-04-01

**Authors:** Michael Cimorelli, Rienk Nieuwland, Zoltán Varga, Edwin van der Pol

**Affiliations:** 1 Department of Chemical & Biological Engineering, Drexel University, Philadelphia, Pennsylvania, United States of America; 2 Department of Clinical Chemistry, University of Amsterdam location AMC, Amsterdam, The Netherlands; 3 Vesicle Observation Center, University of Amsterdam location AMC, Amsterdam, The Netherlands; 4 Department of Biomedical Engineering and Physics, University of Amsterdam location AMC, Amsterdam, The Netherlands; 5 Biological Nanochemistry Research Group, Institute of Materials and Environmental Chemistry, Research Center for Natural Sciences, Budapest, Hungary; University of Houston, UNITED STATES

## Abstract

The particle size distribution (PSD) of extracellular vesicles (EVs) and other submicron particles in biofluids is commonly measured by nanoparticle tracking analysis (NTA) and tunable resistive pulse sensing (TRPS). A new technique for measuring the PSD is microfluidic resistive pulse sensing (MRPS). Because specific guidelines for measuring EVs together with other particles in biofluids with MRPS are lacking, we developed an operating procedure to reproducibly measure the PSD. The PSDs of particles in human plasma, conditioned medium of PC3 prostate cancer cell line (PC3 CM), and human urine were measured with MRPS (nCS1, Spectradyne LLC) to investigate: (i) the optimal diluent that reduces the interfacial tension of the sample while keeping EVs intact, (ii) the lower limit of detection (LoD) of particle size, (iii) the reproducibility of the PSD, (iv) the optimal dilution for measuring the PSD, and (v) the agreement in measured concentration between microfluidic cartridges with overlapping detection ranges. We found that the optimal diluent is 0.1% bovine serum albumin (w/v) in Dulbecco’s phosphate-buffered saline. Based on the shape of the PSD, which is expected to follow a power-law function within the full detection range, we obtained a lower LoD of 75 nm for plasma and PC3 CM and 65 nm for urine. Normalized PSDs are reproducible (R^2^ > 0.950) at dilutions between 10–100x for plasma, 5–20x for PC3 CM, and 2–4x for urine. Furthermore, sample dilution does not impact the dilution-corrected concentration when the microfluidic cartridges are operated within their specified concentration ranges. PSDs from microfluidic cartridges with overlapping detection ranges agreed well (R^2^ > 0.936) and when combined the overall PSD spanned 5 orders of magnitude of measured concentration. Based on these findings, we have developed operating guidelines to reproducibly measure the PSD of EVs together with other particles in biofluids with MRPS.

## Introduction

Extracellular vesicles (EVs) are membrane-enclosed, cell-derived nanoparticles found in biofluids [1]. Research suggests EVs contribute to homeostasis [2–4], disease development and progression [5–7], while showing promise as therapeutic drug delivery vehicles [8–11] and novel biomarkers for disease [12–14]. Reproducible measurements of EV size, shape, function, and concentration are a requirement before researchers can fully understand EV biology and exploit clinical applications of EVs [15]. However, EVs are only a subset of the submicron particles found in most biofluids, which complicates measurements. The particle size distribution (PSD), which describes the relative number of particles in a range of sizes, of EVs in human blood plasma has only been measured by cryogenic electron microscopy and in part by flow cytometry [1, 16] because other detection techniques, such as non-fluorescence nanoparticle tracking analysis (NTA) and tunable resistive pulse sensing (TRPS), are unable to differentiate EVs from other particles. Although NTA and TRPS cannot identify EVs, PSDs and particle number concentrations measured with dedicated sizing techniques are essential to EV science, especially to (i) confirm the presence of submicron particles, (ii) estimate the particle number concentration within the detected size range, (iii) understand potential artifacts of other detection techniques, such as swarm detection for flow cytometry [17], (iv) develop reference and test samples, and (v) understand and optimize isolation methods.

An alternative, novel technique to measure the PSD is microfluidic resistive pulse sensing (MRPS), which is employed in the nCS1 developed by Spectradyne LLC (Torrance, CA, USA) [18]. Similar to NTA and TRPS, MRPS cannot differentiate between EVs and other particles. Previously, we showed that MRPS is able to measure the PSD of red blood cell-derived EVs [19]. However, a detailed standard operating procedure for measuring the PSDs of EVs together with other particles in biofluids by MRPS is absent, we aimed to establish a reliable operating procedure to ensure reproducible PSD measurements with MRPS.

**Fig 1:**
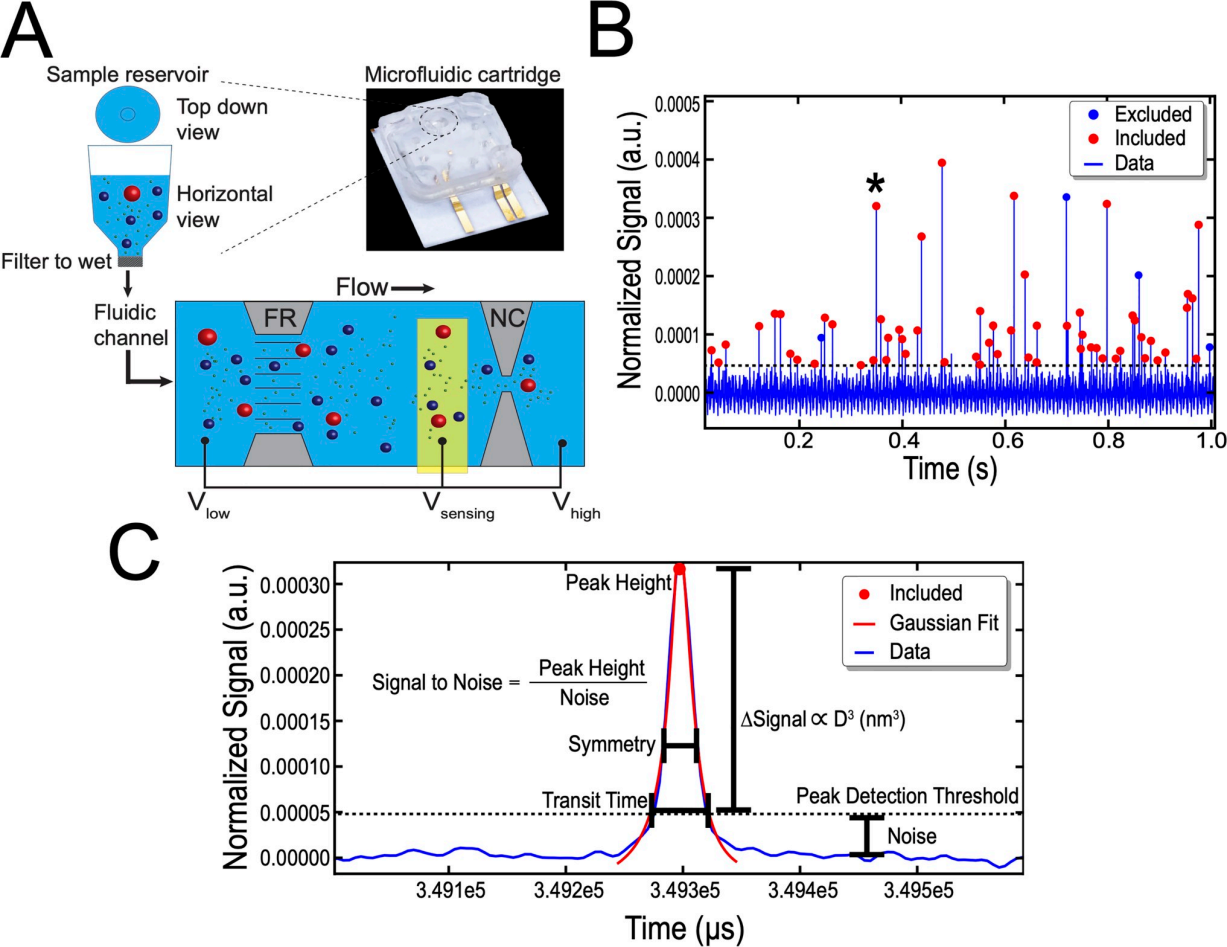
Operating principle of microfluidic resistive pulse sensing (MRPS). (A) Sample is inserted into the sample reservoir of a microfluidic cartridge while ensuring no air bubbles are present. After wetting the pre-filter (‘Filter to wet’ in grey), sample enters the fluidic channel that is biased by external electrodes (V_low_ and V_high_) to generate an ionic electrical current in the sample. A voltage divider is formed by the fluidic resistor (FR) and the nanoconstriction (NC) according to their respective resistances. The sensing electrode (in yellow) is fixed in the microfluidic channel between the fluidic resistor and the nanoconstriction. As a particle enters the nanoconstriction, it lowers the electrical current. The presence of the voltage divider that is formed between the fluidic resistor and the nanoconstriction allows for the measurement of the sensing electrode voltage (V_sensing_). The change in voltage is proportional to the particle volume through the Coulter Principle. (B) Normalized signal (a.u.) versus time (blue line) for particles in plasma measured with a TS-2000 cartridge. The signal is normalized by multiplying by -1, and then dividing the change in voltage during a particle detection event by both the measurement gain and the applied electrical bias; measurement gain and applied electrical bias are pre-determined by the manufacturer and are chip dependent parameters. Data are included (red dots) or excluded (blue dots) based on the manufacturers peak filter recommendations. The peak marked by * is enlarged in panel C. (C) Normalized signal versus time for one particle and a definition of parameters that makeup the peak filters. Data below the peak detection threshold (dotted line) are defined as noise. The peak detection threshold is set by multiplying the standard deviation of the electrical noise by 3. Transit time is defined as the length of time a signal exceeds the peak detection threshold. The nCS1 software fits peaks with a Gaussian function (red line) to determine the symmetry and amplitude of the peak. The symmetry of the peak is used to differentiate between particles and noise. The amplitude of the peak (peak height) is directly related to the diameter of the particle through the Coulter Principle. The ratio of peak height to noise is the signal to noise ratio.

The operating principle of MRPS, which is illustrated in Fig 1, is described in detail by Fraikin et al. [20]. With MRPS, samples are measured inside disposable microfluidic cartridges. Each cartridge has two components: (i) a fluidic channel that guides the sample to the electrical sensing zone by a pressure-driven flow (yellow in Fig 1), and (ii) an electric circuit that measures the change in electrical resistance of the nanoconstriction when particles pass through [20]. Because MRPS is based on the Coulter principle [21], the change in electrical resistance, which is measured as a change in voltage, is proportional to the volume displaced by the particle transiting through the nanoconstriction [22]. In turn, the transit time is used to determine the flow rate, which together with the count rate is used to derive the number concentration.

MRPS differentiates itself from TRPS in that MRPS uses cartridges that have rigid, pre-calibrated pores made of polydimethylsiloxane, while TRPS uses sensing pores of polyurethane that can be stretched to tune the pore size. Although a tunable pore size increases the dynamic range of a pore, the tunability requires pre-calibration and stress relaxation can hamper reproducibility [22, 23]. Also, MRPS controls flow of sample through the nanoconstriction using controlled pressure, limits clogging of the nanoconstriction during measurements using embedded pre-filters, measures flow rates directly from transit time without advance need for calibration, and expands the measurable range of concentrations [24]. A detailed explanation of the differences between MRPS and TRPS can be found in the (see S3 File).

However, several concerns must be addressed to assess the reproducibility of MRPS when measuring the PSD. Due to the design of the cartridges, capillary forces at the interface of the sample reservoir and the subsequent filter prevent the flow of diluted sample into the microfluidic channel (Fig 1A). Therefore, the sample diluent must incorporate a surfactant to act as a wetting agent by lowering the interfacial tension to allow flow into the microfluidic channels. Tween 20, the recommended wetting agent from the manufacturer’s operational manual, is a non-ionic detergent that may lyse EVs [25, 26]. In addition, the manufacturer has specified lower and upper limits of detection (LoD) in terms of particle diameters for each cartridge type. In this work, we will evaluate only the lower LoD of cartridges, because measured particle concentrations in biofluids are mainly affected by the lower LoD due to the skewed PSD. LoD claimed by the manufacturer require scientific validation for submicron particles in biofluids. Therefore, reproducibility of the measured PSD and concentration need to be confirmed as a function of sample dilution, which is independent of the diluent. Because different cartridge molds cover different particle size and concentration ranges, the agreement in the measured concentration between cartridges with overlapping LoD require validation.

We aim to investigate the aforementioned concerns and to develop a methodological procedure to reproducibly measure the PSDs of EVs together with other particles in three commonly studied biofluids: (i) plasma, (ii) conditioned medium of PC3 prostate cancer cell line (PC3 CM), and (iii) urine by MRPS.

## Materials and methods

### Approach

We performed the following five experiments in stepwise order to accomplish our goal. (i) To determine the optimal diluent that reduces the interfacial tension of the sample while keeping EVs intact, we characterized particles in plasma and urine diluted in Dulbecco’s phosphate buffered saline (DPBS), bovine serum albumin (BSA), Tween 20, and Triton X-100 using flow cytometry. Subsequently, we measured the PSDs of diluents when filtered through a 50-nm isopore filter or a 100-kDa centrifugation filter using MRPS. (ii) To define the LoD of a cartridge, we measured the PSDs of plasma, PC3 CM, and urine and compared the measurement with a mathematical function. (iii) To quantify the reproducibility of MRPS in measuring the PSD, we determined Pearson correlation coefficients from the PSD as a function of sample dilution. (iv) To determine the optimal dilution for measuring the PSD, we quantified the relationship between serial dilution and (a) measured concentration and (b) particle counts. (v) Using insights from i-iv, we measured the PSDs of plasma, PC3 CM, and urine using multiple cartridges to quantify the agreement in measured concentration between cartridges with overlapping LoD.

### Biological fluid preparation

Whole blood was collected using a 21G needle in 3-mL citrate Vacutainers (BD Biosciences, San Jose, CA) from 20 healthy participants (10 males, 10 females) with informed consent. Blood was centrifuged at 2,500 g for 15 minutes at room temperature using a Rotina 380R centrifuge (Hettich, Tuttlingen, Germany). The plasma (10 mm above the buffy coat) was transferred to new tubes and centrifuged at 2,500 g for 15 minutes at room temperature to remove residual platelets. Subsequently, the plasma was pooled, and 100-μL aliquots were snap frozen in liquid nitrogen and stored at –80°C until use.

Prostate cancer PC3 cells were cultured at 37°C and 5% CO_2_ in DMEM, RPMI 1640 with L-glutamine (Thermo Fischer Scientific, 11875) supplemented with 10% fetal bovine serum (v/v, FBS), 10 units/mL penicillin, and 10 μg/mL streptomycin. The culture medium was refreshed every other day. Once the cells reached 80 to 90% confluence, the cells were washed three times with DPBS and FBS-free RPMI medium supplemented with 1 unit/mL penicillin and 1 μg/mL streptomycin. After 48 hours, the cell supernatant was collected and centrifuged at 1,000 g for 30 minutes at room temperature in a 1.5-mL Eppendorf tube (Thermo Fischer Scientific) using a Rotina 46RS centrifuge (Hettich, Tuttlingen, Germany). The supernatant was collected and pooled, and 50-μL aliquots were snap frozen in liquid nitrogen and stored at –80°C until use.

Urine was obtained from overnight fasting healthy participants (5 males) with informed consent. The urine was pooled in 8 aliquots of 50 mL and centrifuged twice in 50-mL Greiner tubes, 180 g for 10 minutes at 4°C and 1,560 g for 20 minutes at 4°C, to remove cells using a Rotina 380R centrifuge. The pooled urine was separated into 1-mL aliquots and snap frozen in liquid nitrogen and stored at –80°C until use.

Before analysis, pooled plasma and PC3 CM were thawed for 1 minute at 37°C in a water bath, while pooled urine was thawed for 3 minutes at 37°C to dissolve amorphous salts. All samples were stored on ice until the start of an experiment.

Blood and urine samples were collected with written informed consent in accordance with the Helsinki Declaration and approved by the medical-ethical assessment committee of the Academic Medical Center, University of Amsterdam.

### Diluent selection and preparation for flow cytometry

To measure the PSD with the nCS1, it is necessary that the diluent incorporate a surfactant to wet the filter below the sample reservoir. This allows the diluted sample to flow into the microfluidic channel (Fig 1A). The nCS1 operator’s manual recommends diluting samples with 1% Tween 20 (v/v) in DPBS [18]. However, as Tween 20 may lyse EVs at sufficiently high concentration [25, 26], we used flow cytometry to determine if the measured concentration of stained EVs in plasma or urine are affected in the presence of three surfactants in DPBS: (i) 0.01% and 1% Tween 20 (v/v), (ii) 0.1% BSA (w/v), and (iii) 0.1% Triton X-100 (v/v). Triton X-100 serves as a positive control as it is commonly used in cell-lysis assays [25, 27]. The lower concentration of Tween 20 (0.01% v/v) was chosen as this is the critical micelle concentration (CMC) of Tween 20 at room temperature. The CMC of a surfactant is defined as the concentration above which any added surfactant molecules aggregate into micelles, and as a consequence, the interfacial tension of the solution remains constant upon addition of more surfactant [28]. At the CMC, interfacial tension is minimized without the use of excess surfactant. Stained EVs in plasma or urine were also diluted with DPBS without a surfactant as a negative control.

To determine the concentrations of stained EVs in plasma and urine and of unstained particles in the diluent, such as micelles or protein aggregates, all samples were analyzed by flow cytometry (A60-Micro, Apogee Flow Systems, Northwood, UK) at a flow rate of 3.01 μL/min. Flow cytometry was the preferred method to determine if sample diluent affects the measured concentration of EVs in plasma or urine because flow cytometry, unlike MRPS, is able to differentiate stained EVs from unstained non-EV particles. The reported concentrations are from EVs that exceeded the side scatter threshold (10 nm^2^), had a diameter > 200 nm but < 800 nm as determined by Flow-SR [16], had a refractive index < 1.42 to exclude chylomicrons [29], and were positive at the fluorescence detector corresponding to the fluorophore used. Flow-SR is based on the ratio of side and forward light scattering signals. Because the forward light scattering signal has limited sensitivity, the lower limit of our analysis, in terms of particle diameter, is 200 nm.

We labeled EVs in plasma with both lactadherin-fluorescein isothiocyanate (FITC) and CD61-allophycocyanin (APC) and EVs in urine with lactadherin-FITC. Our experiment followed the newly established guidelines for reporting EV flow cytometry experiments (MIFlowCyt-EV) [30], thus we calibrated all detectors, quantified the EV diameter and refractive index from the flow cytometry scatter ratio (Flow-SR) [16], and used custom developed software (MATLAB 2018b, MathWorks, USA) to automate data analysis and processing. All details about the sample staining protocol, instrument calibration, experimental settings, data acquistion, and characterization of EVs are included in the supplemented MIFlowCyt-EV (see S1 File).

### nCS1 measurements

MRPS measurements were performed using an nCS1 with hardware version 0. The micofluidic system is primed with a solution of 1% Tween 20 (v/v) in DPBS. Priming is the process of generating an appropriate ionic electric current in the system and wetting all channels of the cartridge. After priming, the pressures inside the microfluidic cartridge are set to establish a direction of fluid flow that ensures the diluted biofluid does not make contact with the 1% Tween 20 (v/v) prior to analysis in the nanoconstriction. Therefore, there is no risk of membrane lysis by Tween 20 [18, 20]. The instrument parameters, specifically the pressure at each port of the cartridge and the voltages at each bias electrode, were not controlled manually, but determined automatically by the device. The pressure at each port of the cartridge were: P1 IN = 3.5 psig, P5 OUT = 3.0 psig, P3 IN = 3.0 psig, P2 OUT = 2.0 psig, P7 IN = 3.0 psig, and P6 OUT = 2.4 psig [18, 20]. The flow topology of the cartridges is not public, however, the pressure at each port of the cartridge is explained in prior iterations by Fraikin et al. [20]. The voltages of the two bias electrodes are specific and optimized for each type of cartridge (whether a TS-300 vs TS-400) and the lot number of the cartridge (mold-ID). For reference, the three cartridge mold-IDs used throughout this work were P12, P15, or P27 and these mold IDs correspond to bias voltage values of -4/+5 V, -5/+6 V, or -2/+2 V, respectively.

To prepare a clean diluent that does not generate false-positive counts, we investigated two filtration methods: (i) a single pass through a 50-nm isopore filter (Merck Millipore, Burlington, MA, USA) and (ii) a Vivaspin 500, 100-kDa centrifugation filter (Merck Millipore, Burlington, MA, USA) operated at 12,000 g for 5 minutes at room temperature. The filtration methods were compared by measuring the PSDs of both diluents with MRPS to reveal the filtration method that resulted in the lowest concentration of background counts.

To determine the LoD and to investigate the relationship between sample dilution, acquisition time, and measured concentration and particle counts, each biofluid was studied with a cartridge according to recommendations from the manufacturer to minimize the frequency of pore clogging. TS-300 cartridges (50 to 300 nm) were used to determine the PSD of particles in urine, while TS-400 cartridges (65 to 400 nm) were used to determine the PSD of particles in plasma and PC3 CM. For each measurement, 5 μL of diluted sample was used. All samples were diluted such that the resulting particle concentrations fell within the operational range of each cartridge specified by the manufacturer. All experiments consisted of 10 acquisitions of 10 seconds (100 s of data), and experiments were repeated in triplicate with three new/separate cartridges to quantify cartridge to cartridge variability.

After a measurement was completed, data were combined and (i) peak filters and (ii) background subtraction were applied, as recommended by the manufacturer, in the nCS1 Data Viewer (Version 2.5.0.249) [18]. The peak filters were applied to individually detected particles, and the parameters that make up the peak filters are defined in Fig 1C. The peak filters used in this work are: Transit time (μs) < 60, Symmetry > 0.2 but < 4.0, Diameter (nm)—set by cartridge (TS-300 > 50 nm; TS-400 > 65 nm; TS-2000 > 250 nm), Signal to noise ratio (S/N) > 10. The peak filters were recommended by the manufacturer and independently confirmed as appropriate by determining the mean transit time, symmetry, and signal to noise ratio of a single dataset of particles found in plasma with greater than > 10,000 particle detection events. To avoid false positive counts due to electrical noise, we applied background subtraction to all measurements. The brief explanation of the algorithm behind background subtraction is added to the (see S2 File).

After three independent measurements were completed, PSDs were exported from the nCS1 Data Viewer (Version 2.5.0.249) with 10-nm bins. Post processing was done in Python (v.3.8.0), and graphing was done in GraphPad Prism 8 (GraphPad Software, San Diego, CA, USA). We determined the LoD by assuming that the measured PSD follows a power-law function within the detection range of the cartridges used. For urine, this assumption was confirmed earlier, as the PSD follows a power-law function down to 50 nm [31]. We define the LoD as the minimum diameter for which the measured PSD follows the power-law function within 50% relative error. Three independent measurements were averaged to quantify (i) concentration, (ii) particle counts, and (iii) coefficients of determination (R^2^) of the PSDs.

Subsequently, we measured the PSDs of plasma and PC3 CM with TS-400 and TS-2000 cartridges and of urine with TS-300, TS-400, and TS-2000 cartridges to quantify the agreement in measured concentration between multiple cartridges with overlapping LoD [18]. To handle data in the overlapping regions of the PSD from multiple cartridges, we averaged the measured concentrations from the cartridges and propagated the error to correspond with 95% confidence intervals. The MRPS data collected during this study and discussed herein can be found at *DOI*: 10.6084/m9.figshare.14100995.

## Results

**Fig 2:**
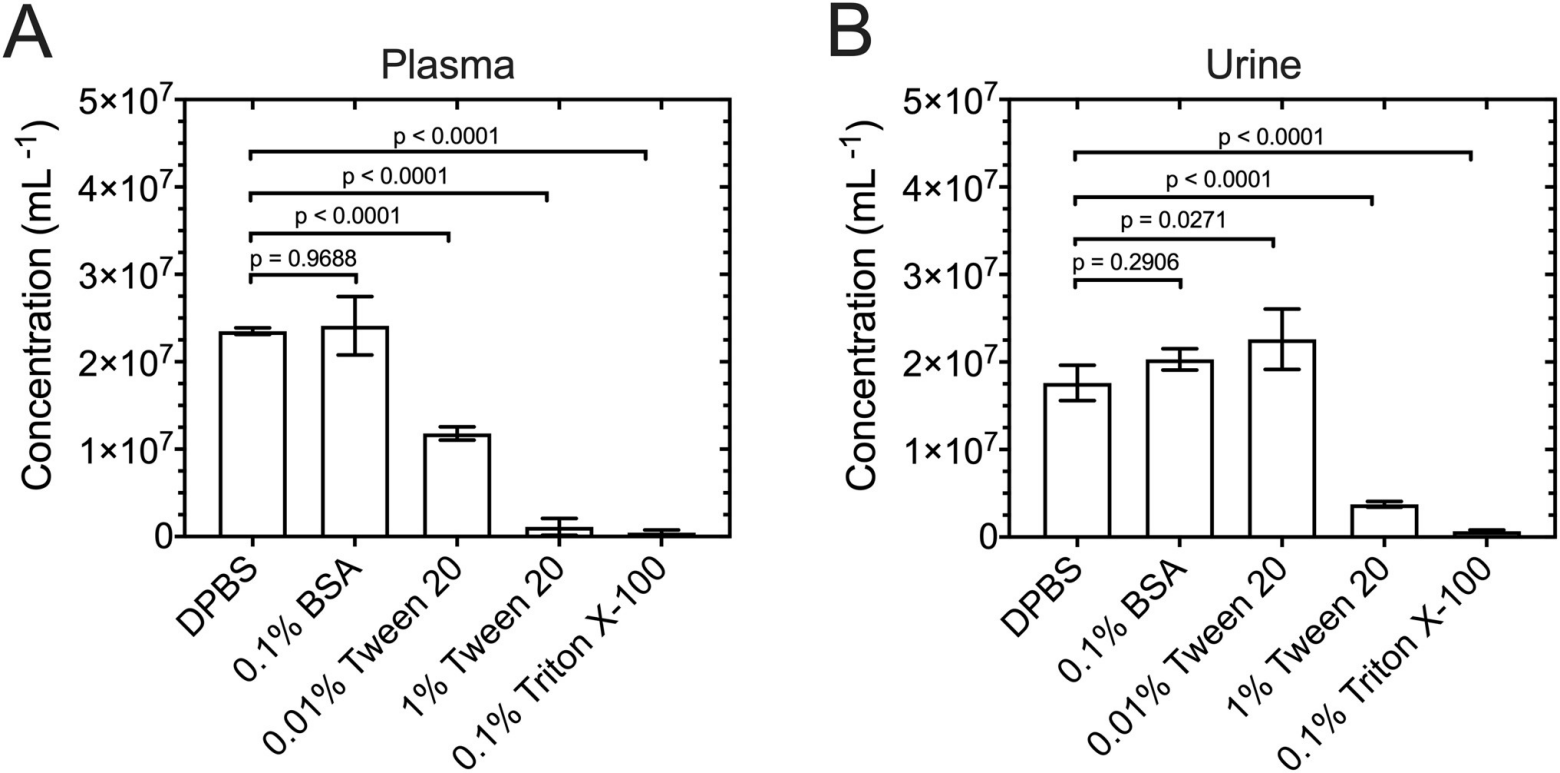
Motive to dilute EVs in BSA as opposed to Tween 20 for measurements with microfluidic resistive pulse sensing (MRPS). Concentration of (A) CD61-APC+ > 150 MESF) and lactadherin-FITC+ > 350 MESF) EVs in plasma and (B) lactadherin-FITC+ > 650 MESF) EVs in urine diluted in DPBS, 0.1% BSA (w/v), 0.01% Tween 20 (v/v), 1% Tween 20 (v/v), and 0.1% Triton X-100 (v/v) measured by flow cytometry. Based on Flow-SR, EV diameters ranged from 200 nm to 800 nm and EV refractive indices were < 1.42 (gates in S1 File). Error bars are representative of sample standard deviations (n = 3). Statistical significance was quantified with a one-way ANOVA comparing the means against DPBS (control). P values were adjusted for multiple comparisons using a Dunnett’s test.

**Fig 3:**
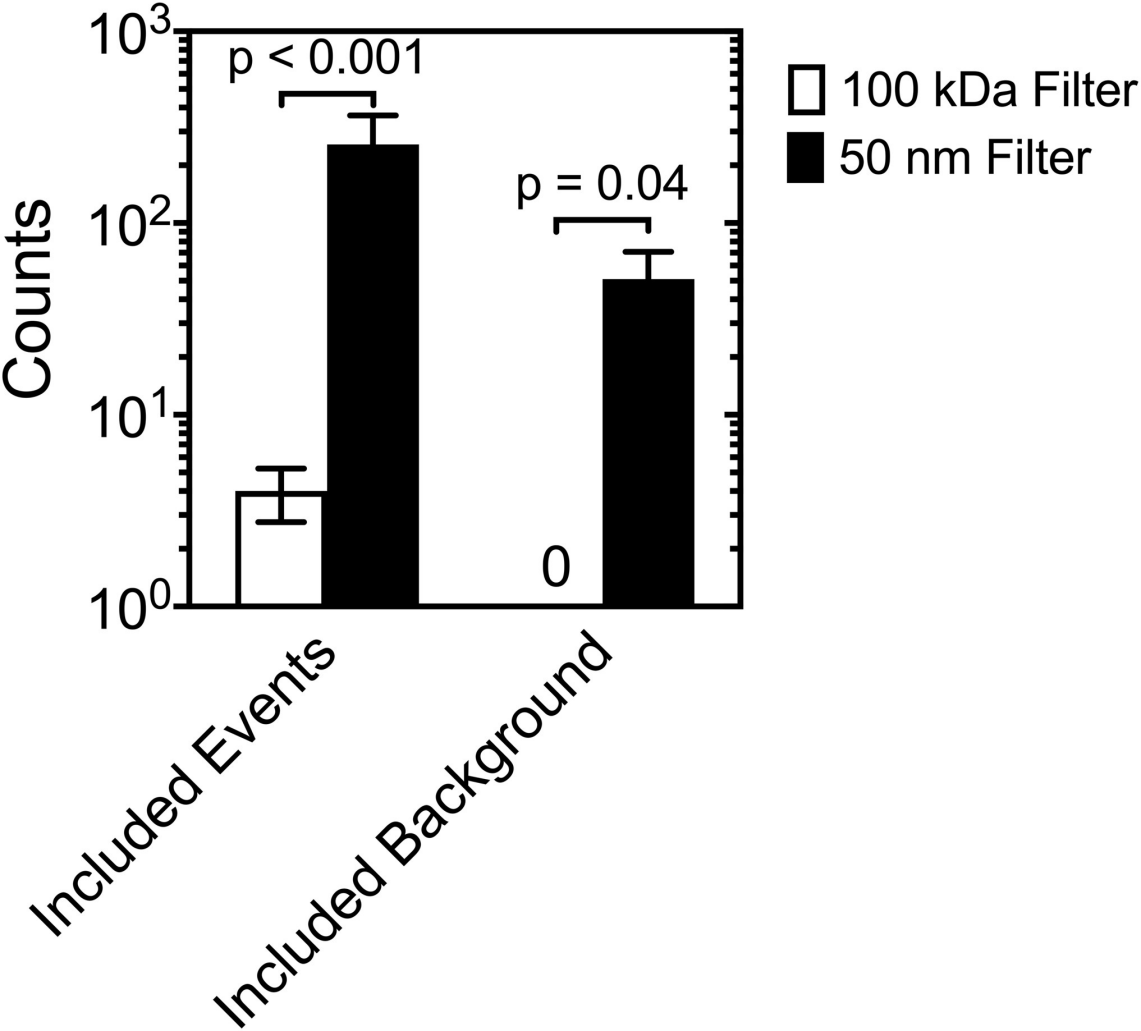
Motive to filter BSA with a 100 kDa filter to reduce false positive background counts for measurements with microfluidic resistive pulse sensing. Counts from 0.1% BSA (w/v) in DPBS prepared by a 100-kDa filter and a 50-nm Isopore filter in a TS-300 microfluidic cartridge. Data were averaged after three independent measurements, of which each consisted of 10 acquisitions of 10 seconds. The background counts were subtracted, and the peak filters were applied. Included events are particle detection events that pass the peak filters, while Included background is electrical noise that is included as a particle detection event by passing the peak filters. Error bars are representative of 95% confidence intervals (n = 3). Statistical significance was quantified with a two-way ANOVA; p values were adjusted for multiple comparisons using a Sidak test.

To find the optimal diluent, Fig 2 shows the concentration of (A) CD61-APC+ (> 150 MESF) and lactadherin-FITC+ > 350 MESF) EVs in plasma and (B) lactadherin-FITC+ > 650 MESF) EVs in urine diluted in 0.01% and 1% Tween 20 (v/v), 0.1% BSA (w/v), 0.1% Triton X-100 (v/v) (positive control), and DPBS (negative control) measured with flow cytometry. Both plasma and urine in 0.1% BSA (w/v) resulted in similar EV concentrations as DPBS (p = 0.9688 for plasma and p = 0.2906 for urine). Plasma in 0.01% and 1% Tween 20 (v/v) or 0.1% Triton X-100 (v/v) resulted in significantly lower EV concentrations than DPBS (p < 0.0001). Urine in 0.01% Tween 20 (v/v) resulted in an increase in EV concentrations relative to DPBS (p = 0.027), while concentrations of 1% Tween 20 (v/v) and 0.1% Triton X-100 resulted in significantly lower EV concentrations than DPBS (p < 0.0001). MRPS measurements with a TS-300 cartridge revealed that filtering 0.1% BSA (w/v) in DPBS with a 100-kDa centrifugation filter had 4 ± 1 (n = 3) particle detection events and 0 ± 1 (n = 3) false positive background counts in 100 s of data, while a single pass through a 50-nm isopore filter had 243 ± 43 (n = 3) and 51 ± 8 (n = 3) false positive background counts in 100 s of data as shown in Fig 3. Therefore, all samples in this manuscript were diluted in 0.1% BSA (w/v) in DPBS and filtered with a 100-kDa centrifugation filter.

**Fig 4:**
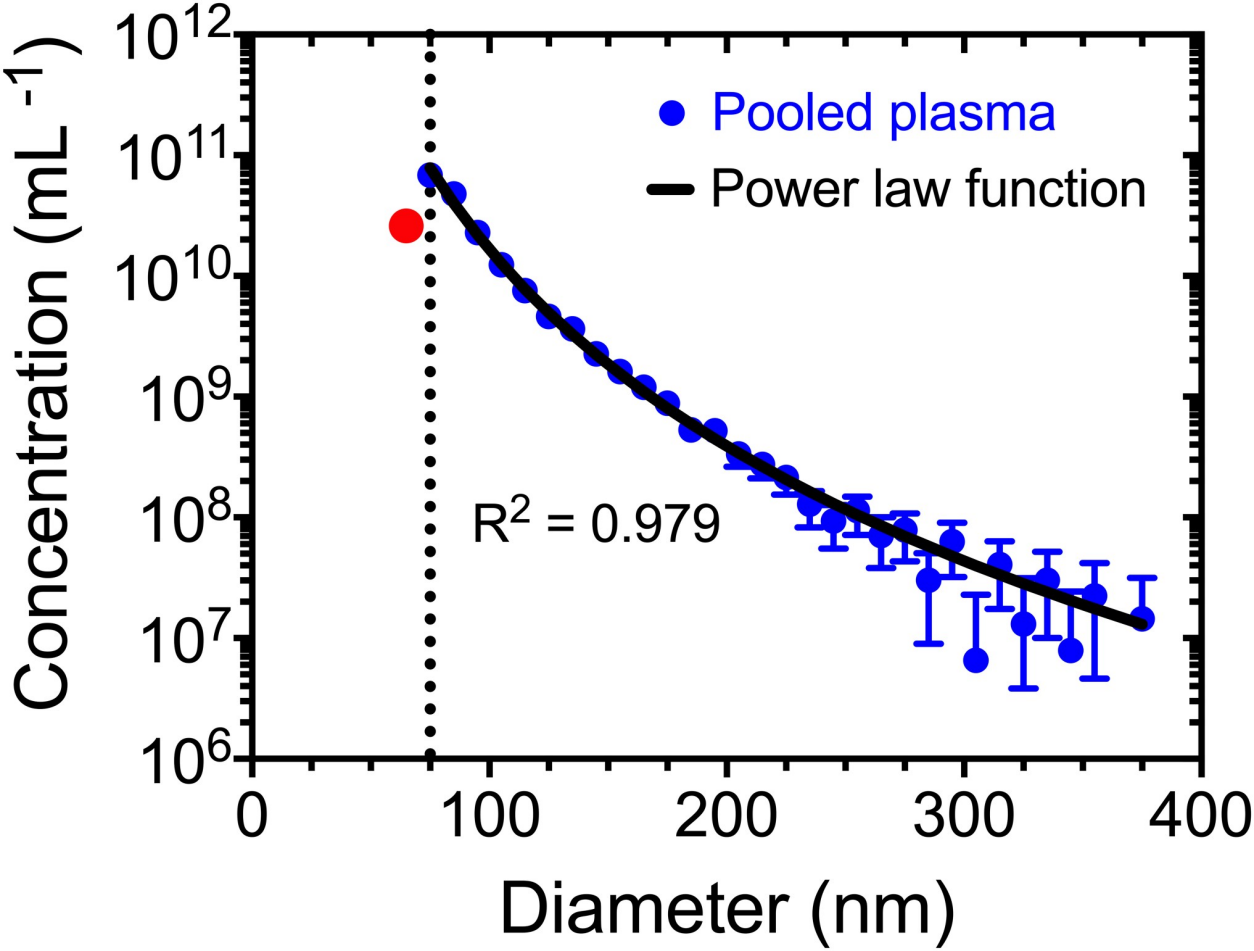
The detection limit of a TS-400 is approximately 75 nm when measuring plasma diluted 10-fold. Concentration of EVs together with other particles found in 10-fold diluted plasma versus diameter (10-nm bins) measured with microfluidic resistive pulse sensing using a TS-400 cartridge. Data were averaged after three independent measurements, of which each consisted of 10 acquisitions of 10 seconds. The background counts were subtracted, and the peak filters were applied. The measured particle concentration at 65 nm (red circle) was below the detection limit (dotted line), therefore omitted for fitting. Error bars represent 95% confidence intervals (n = 3). For datapoints without visible error bars, the error bars are smaller than the symbol. The power law fit parameters (*a* = 1.084 × 10^21^ and *k* = 5.409) were determined by applying a linear regression to the logarithm of the concentration and diameter.

To quantify the LoD per cartridge, Fig 4 shows the PSD of plasma measured by a TS-400 cartridge from 65–375 nm in 10-nm bins. The data follow a power-law function (R^2^ = 0.979) over approximately four orders of magnitude concentration. Under the assumption that the power-law function extends to approximately 50 nm [31], the measured concentration at 65 nm, denoted by the red circle, is more than 50% different than the power-law function. Therefore, the detection limit was estimated to be 75 nm, denoted by the dotted line. This procedure was repeated and applied to all samples to estimate the LoD.

**Fig 5:**
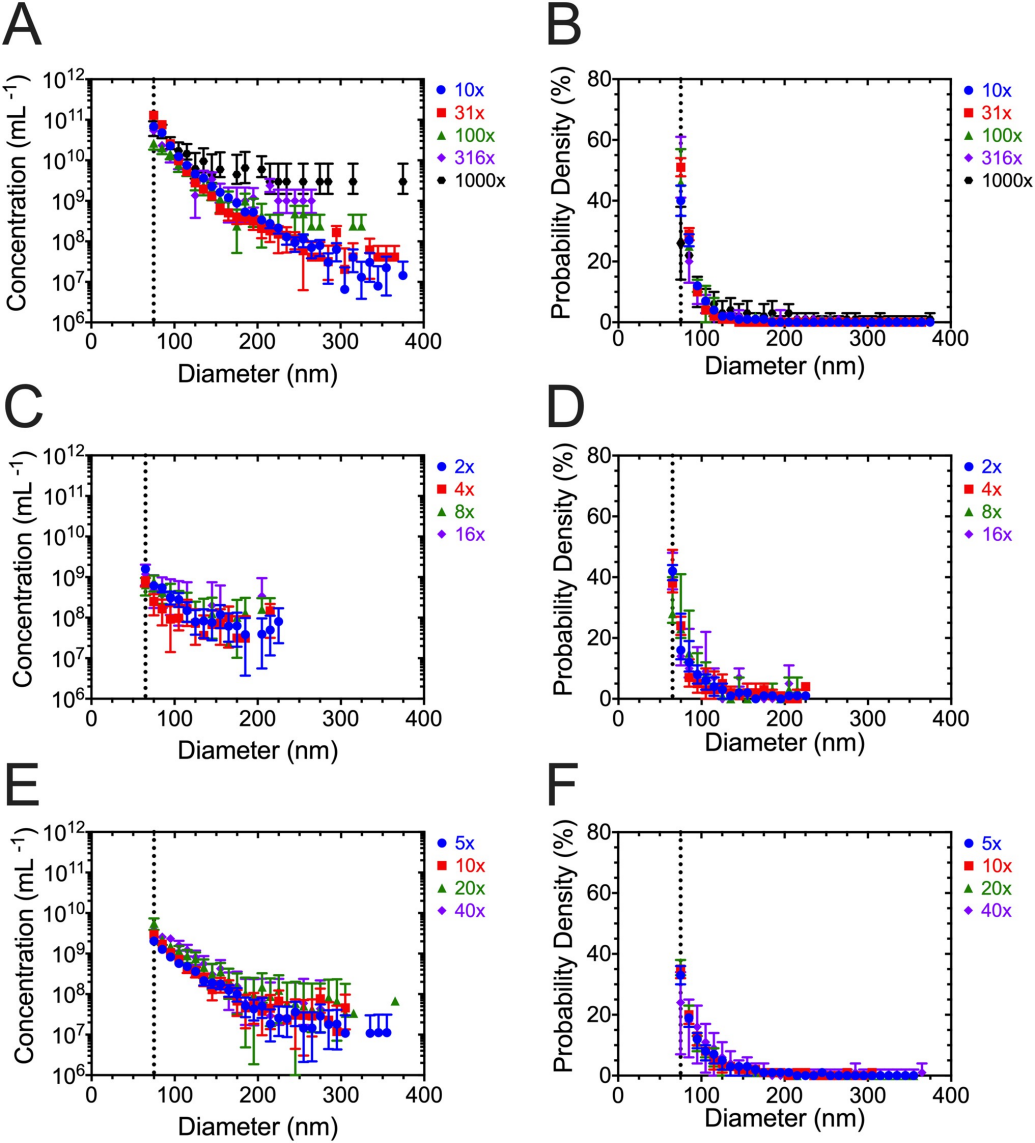
The measured particle size distribution is independent of sample dilution. The average measured concentration corrected for dilution of (A) plasma, (C) PC3 CM, and (E) urine as a function of diameter (10-nm bins) after removing the first bin of data. The average probability density from serial dilutions of (B) plasma, (D) PC3 CM and (F) urine as a function of diameter (10-nm bins) after removing the first bin of data. Data were averaged after three independent measurements, of which each consisted of 10 acquisitions of 10 seconds. The peak filters were applied. Error bars represent 95% confidence intervals (n = 3). The dotted line at 75 nm in A-D, and 65 nm in E-F denote the detection limit after removing the first bin.

To investigate the reproducibility of the measured PSD as a function of dilution, Fig 5 shows the measured PSDs normalized to number concentration (corrected for dilution) and the PSDs as probability density functions for plasma, PC3 CM, and urine. We obtained a LoD of the TS-300 and TS-400 cartridge of 65 nm and 75 nm, respectively. The LoD was independent of sample dilution. In Fig 5A and 5C, samples measured at the highest dilutions seem to overestimate the measured concentration. However, this apparent overestimation is merely an artifact caused by the stochastic nature of particles transiting through the nanoconstriction. Because the number of particles counted per bin decreases as the dilution factor increases, the stochastic detection of one particle multiplied by the dilution factor results in an apparent overestimation of the measured concentration, whereas the stochastic absence of one particle results in an apparent concentration of 0 mL^−1^. The bins with a concentration of 0 mL^−1^, however, are not visible due to logarithmic data representation. Fig 5B, 5D and 5F illustrate that the probability density of the measured diameters is independent of sample dilution, thereby confirming that the overestimation is a statistical error (see Discussion). Table 1 shows that the PSDs had a high level of agreement (R^2^ > 0.895 for all dilutions) for plasma, PC3 CM, and urine, regardless of the different dilutions.

**Table 1:** Coefficients of determination (R^2^) determined from Pearson correlation coefficients for plasma, PC3 CM, and urine.

Plasma						PC3 CM					Urine				
	10x	31x	100x	316x	1000x		5x	10x	20x	40x		2x	4x	8x	16x
10x	1.000					5x	1.000				2x	1.000			
31x	0.990	1.000				10x	0.995	1.000			4x	0.950	1.000		
100x	0.993	0.995	1.000			20x	0.995	0.995	1.000		8x	0.920	0.919	1.000	
316x	0.967	0.986	0.987	1.000		40x	0.960	0.948	0.943	1.000	16x	0.970	0.909	0.895	1.000
1000x	0.979	0.952	0.961	0.917	1.000										

**Fig 6:**
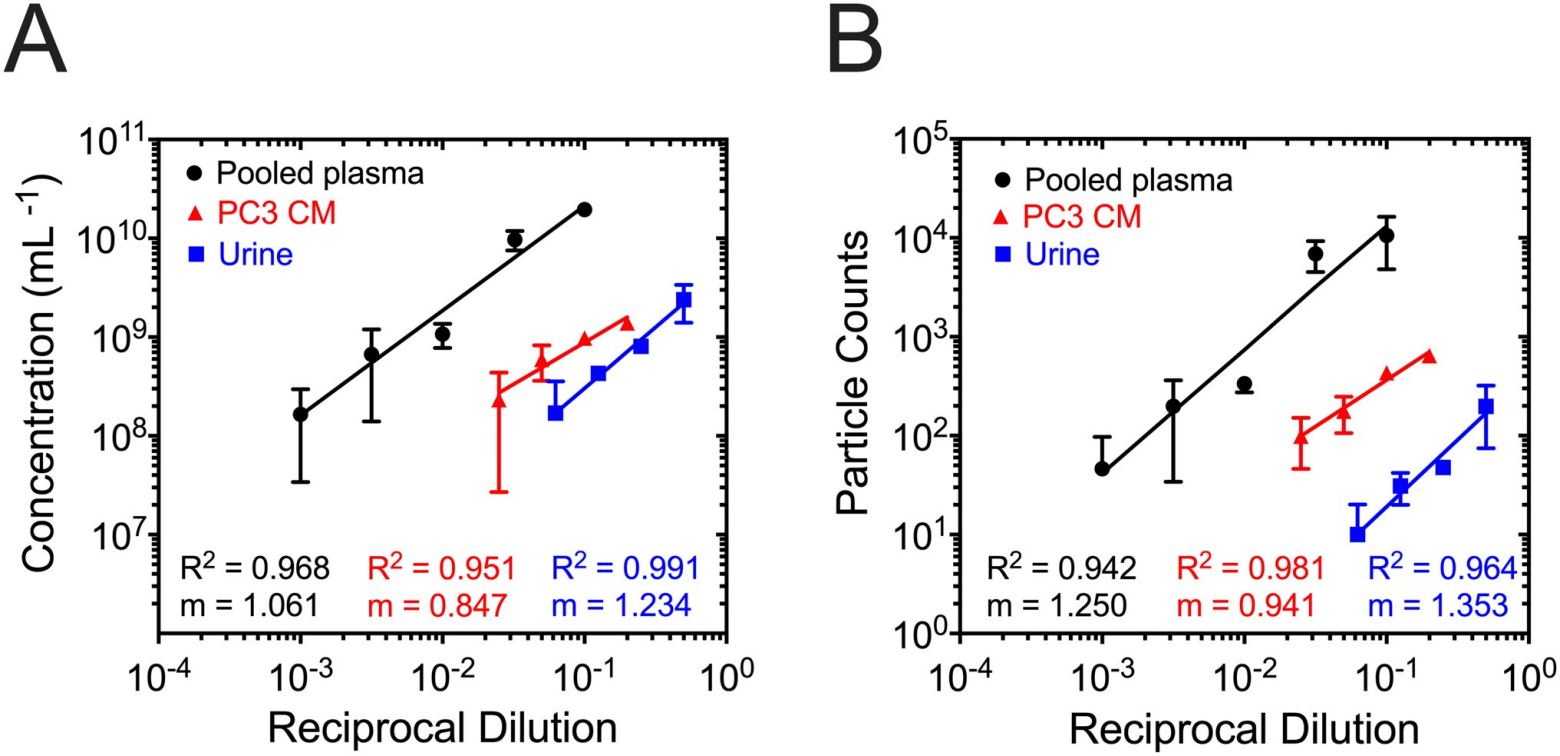
Relationship between measured concentration and particle counts as a function of reciprocal dilution is linear. (A) Average measured concentration and (B) average particle counts in 100 s of acquired data versus reciprocal dilution of plasma (TS-400 cartridge), PC3 CM (TS-400 cartridge), and urine (TS-300 cartridge) after subtracting the electrical noise and applying peak filters. The slope of the regression line for measured concentration is less than the slope for particle counts for all samples studied as the total volume that passes through the nanoconstriction (sample and diluent) is not constant between measurements. Error bars represent the standard deviation (n = 3); when the error bars are not visible, error is smaller than the symbol size. The fit parameters were determined by applying a linear regression to the logarithm of the concentration and reciprocal dilution. The constants of the linear regression for plasma concentration and particle counts are: *m* = 1.061 and *b* = 11.390 and *m* = 1.250 and *b* = 5.368, respectively. The constants of the linear regression for PC3 CM concentration and particle counts are: *m* = 0.847 and *b* = 9.79 and *m* = 0.941 and *b* = 3.503, respectively. The constants of the linear regression for urine concentration and particle counts are: *m* = 1.234 and *b* = 9.72 and *m* = 1.353 and *b* = 2.634, respectively.

To investigate whether dilution affects the measured concentration, Fig 6A shows the measured concentration versus reciprocal dilution for plasma (TS-400), PC3 CM (TS-400), and urine (TS-300). Because for all samples the average particle concentration versus reciprocal dilutions follows a linear function with a slope close to 1.0, the (dilution corrected) concentration measured by MRPS is independent of sample dilution. Fig 6B shows the average particle counts as a function of reciprocal dilution, which also follows a linear trend. However, the slopes differ between samples and exceed 1.0 as the flow rate differs between cartridges and measurements.

**Fig 7:**
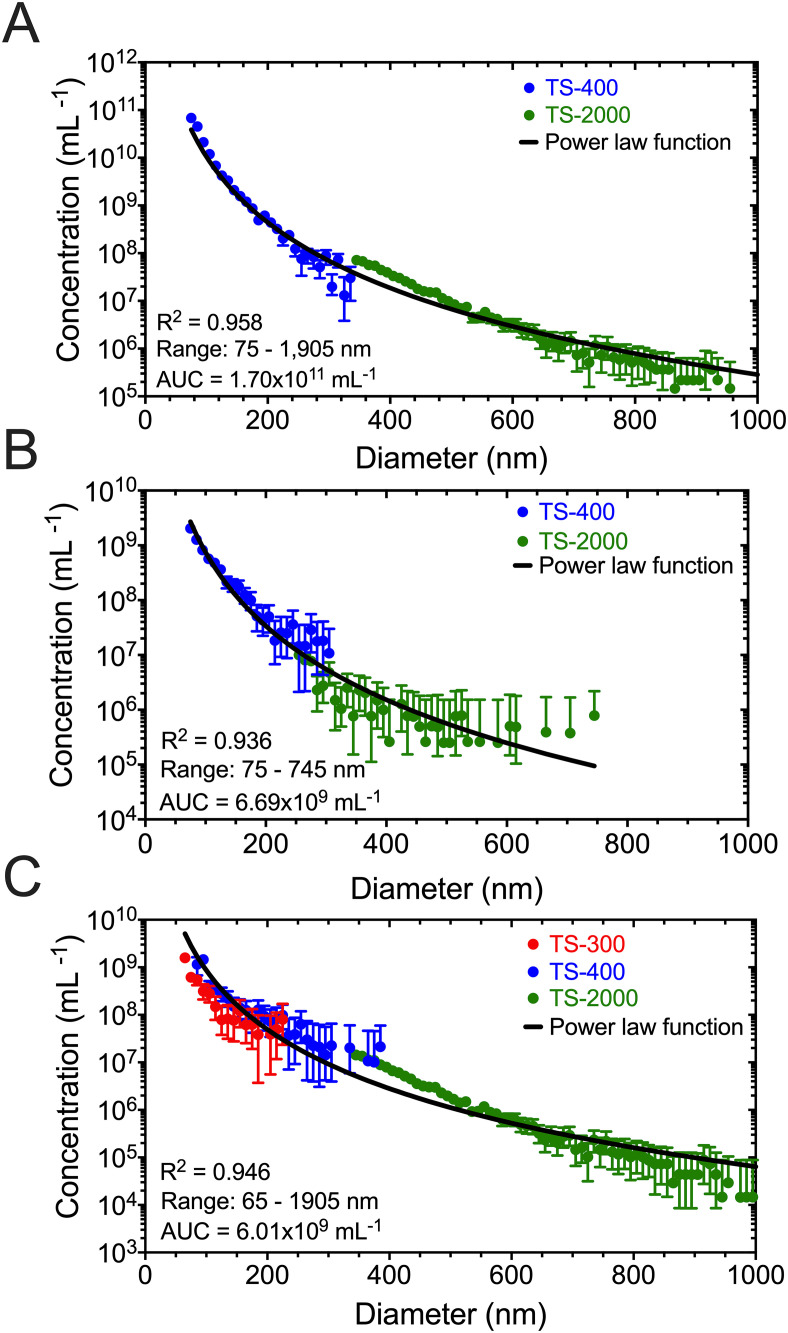
Overlaying multiple cartridges allows for broad particle size determination. Concentration of particles found in (A) plasma, (B) PC3 CM, and (C) urine versus diameter (10-nm bins) measured with microfluidic resistive pulse sensing. Plasma and PC3 CM were measured with a TS-400 and TS-2000 cartridge, while urine was measured with a TS-300, TS-400, and TS-2000 cartridge. Data were averaged after three independent measurements, of which each consisted of 10 acquisitions of 10 seconds. Error bars are representative of 95% confidence intervals (n = 3); when the error bars are not visible, error is smaller than the symbol size. The power law fit parameters were determined by applying a linear regression to the logarithm of the concentration and diameter. The coefficient of determination (R^2^), dynamic range studied, and area under curve (AUC) are listed in the bottom-left corner of (A), (B), and (C). The constants of the power law function for plasma are: *a* = 1.405 × 10^19^ and *k* = 4.564. The constants of the power law function for PC3 CM are: *a* = 6.156 × 10^17^ and *k* = 4.463. The constants of the power law function for urine are: *a* = 1.544 × 10^17^ and *k* = 4.128.

To investigate the agreement in measured concentration between cartridges with overlapping LoD, we measured the PSDs of plasma, PC3 CM, and urine with multiple cartridges to cover a specified size range of 65 nm to 2 μm (plasma and PC3 CM) or 50 nm to 2 μm (urine). Fig 7A–7C illustrates the PSD of particles in (A) plasma, (B) PC3 CM, and (C) urine over a broad dynamic range. The most concentrated sample is plasma followed by PC3 CM and urine. There is a strong agreement in the measured PSDs and power-law functions for plasma (R^2^ = 0.958), PC3 CM (R^2^ = 0.936), and urine (R^2^ = 0.946), highlighting how well the power-law function describes PSDs resulting from combined measurements with different cartridges [31]. This finding (i) displays the reproducibility of MRPS to determine both particle size and concentration, and (ii) validates our assumption that particles in plasma, PC3 CM, and urine follow a power-law function for more than 5 orders of magnitude of the measured concentration and down to the LoD of the used cartridges.

## Discussion

In this study, we demonstrated that MRPS is able to reproducibly measure the PSD of EVs together with other particles in biofluids down to 65–75 nm. Specifically, we have developed an operating procedure, found in Table 2, to perform reproducible MRPS measurements with commonly studied biofluids. We determined the optimal diluent, settings, LoD, and sample dilution, while quantifying the reproducibility of the measured PSD, and the agreement in measured concentration between multiple cartridges.

**Table 2:** Operating guidelines to reproducibly measure the particle size distribution of EVs together with other particles in biofluids with microfluidic resistive pulse sensing.

Steps	Operating procedure
*1.0*	*Prepare and filter 0.1% BSA (w/v) in DPBS buffer*
1.1	Filter deionized water with 100 kDa centrifugation filter at 12,000 RPM for 5 minutes to remove trace amounts of sodium azide and glycerol
1.2	Filter 0.1% BSA (w/v) in DPBS buffer with 100 kDa centrifugation filter at 12,000 RPM ≥ 5 minutes
*2.0*	*Dilute biofluid with 0.1% BSA (w/v) to be near the middle of the specified concentration* *range of cartridge chosen*
2.1	For plasma dilute 50-100 fold, PC3 CM dilute 5-10 fold, urine 2-4 fold
2.2	If biofluid concentration is unknown, rely on orthogonal techniques or literature values to estimate the dilution
*3.0*	*Operate nCS1 according to manufacturer*’s manual. Below are additional steps and *important details*
3.1	Pipette 5 μl of diluted sample into sample reservoir and open nCS1 software
3.2	Ensure when pipetting that no air bubbles are between the sample and the pre-filter in the reservoir
3.3	Lightly tap the cartridge on a tabletop to remove any air bubbles that could be present
*4.0*	*Insert cartridge into nCS1 and begin acquisitions until sufficient particles are counted*
4.1	To report a sample concentration, count until ≥ 1,000 events (3.2% counting error)
4.2	To report a particle size distribution, the number of counts depend on the profile of the particle size distribution and required significance per bin
*5.0*	*Filter and export data in Data Viewer Software*
5.1	Combine acquisitions in *Data Viewer Software*
5.2	Apply peak filters to combined file – Transit time (μs) < 60, Symmetry > 0.2 but < 4.0, Diameter (nm)—set by cartridge, Signal to noise ratio (S/N) > 10
5.3	Subtract background to remove particle events accredited to noise
5.4	Export data
*6.0*	*Analyze data to determine detection limit and quantify concentration and particle counts*
6.1	If typical EV sample, fit PSD with power-law function to determine limit of detection
6.2	Under the assumption that the power-law extends below or to the detection limit of the nCS1, set the detection limit when the measured concentration is underestimated by the power-law function
6.3	If the PSD cannot be described by a power-law function, set the detection limit at the onset of measurable electrical noise from the subtract background function

Regarding the diluent, we investigated the effect that 0.1% BSA (w/v), 0.01% or 1.0% Tween 20 (v/v), and 0.1% Triton X-100 (v/v) have on the EV concentration in plasma and urine with flow cytometry. We found that 0.1% BSA (w/v) did not affect the measured concentration of EVs in plasma or urine (Fig 2). As albumin is the most abundant protein in plasma, we expected that BSA would not lyse EVs or hamper the binding of commonly used antibodies or proteins. We found that both 0.01% and 1% Tween 20 (v/v) reduces the measured concentration of EVs in plasma, like Triton X-100, a commonly used detergent for phospholipid membrane lysis [32, 33]. However, in urine, we found that 0.01% Tween 20 slightly increases the measured concentration of EVs, while both 1% Tween 20 (v/v) and 0.1% Triton X-100 (v/v) significantly reduces the measured concentration of EVs. After the addition of 0.01% or 1% Tween 20 (v/v), there is a decrease in the measured concentration of EVs in plasma with flow cytometry, and we propose three plausible explanations. Tween 20 may (i) break apart aggregates that have an effective diameter >200 nm, (ii) lyse EVs, or (iii) interfere with antibody labeling. Other researchers have shown that 1% Tween 20 (v/v) does not affect the measured concentration of EVs with flow cytometry [25]. Hence, future research is needed to elucidate if Tween 20 breaks apart aggregates, lyses EVs, or interferes with the binding of antibodies (used in this work). Ultimately, for EV researchers it is imperative to prevent EV lysis or alteration of the surface morphology of EVs by using Tween 20 when safe alternatives, such as BSA, are available. Therefore, to allow for seamless comparison with orthogonal techniques as recommended by MISEV, we recommend diluting all biofluids in 0.1% BSA (w/v) in DPBS when performing MRPS measurements. To keep the noise floor below the LoD of a TS-300 cartridge, we recommend preparing BSA using a 100-kDa centrifugation filter as opposed to a single pass through a 50-nm isopore filter (Fig 3).

For many biofluids, the LoD of a cartridge can be assessed by analyzing the measured PSD and fitting the data with a power-law function. Our approach to estimate the LoD relies on the assumption that the PSD follows a power-law function. In Fig 4, the measured concentration at 65 nm (red circle) is overestimated by the power-law function due to swarm detection, an increase in the noise floor, or combinations of both. Similar to flow cytometry, swarm detection may occur in MRPS when multiple particles at or below the LoD simultaneously pass through the nanoconstriction, thereby increasing the noise floor, increasing the LoD, and decreasing the reported concentration. Due to the power-law function of the PSD, biofluids typically have particles below the LoD that may cause swarm detection [17]. Another consequence of swarm detection and an increased noise floor is that the LoD will differ from the manufacturer’s specified LoD.

For correct interpretation of data, we recommend identifying the LoD at the point when the measured PSD of biofluids deviate from the power-law function. If 10-nm bins are used, we recommend omitting the first bin of the measured PSD, as most particles belonging to the first bin are below the estimated LoD. However, if smaller bin sizes are used, we recommend fitting the measured PSD with a power-law function to quantify when the measured PSD is less than 50% of the value reported by the power-law function. If the PSD cannot be described by a power-law function, estimate the LoD from the onset of measurable electrical noise from the subtract background function.

To quantify the reproducibility of MRPS, we (i) compared the PSD normalized to number concentration (probability densities) and (ii) calculated R^2^ (Table 1) of PSDs from serial dilutions of plasma, PC3 CM, and urine (Fig 5B, 5D and 5F). Probability densities were calculated from three measurements, each with a new cartridge, after removing the first bin of data that was under-reported. Thus, the PSDs shown in Fig 5 have two key highlights: (i) measured PSDs are reproducible regardless of dilution, which is confirmed by R^2^ > 0.895, and (ii) low inter-cartridge variability at measuring the PSD, which is apparent in the overlapping 95% confidence intervals. Although the agreement between PSDs corrected for dilution (Fig 5A, 5C and 5F) seems to decrease with increasing dilution, this is merely a statistical artifact. At high dilutions, the stochastic detection of one particle multiplied by the dilution factor results in an apparent overestimation of the measured concentration of particles for the corresponding bin. The R^2^ > 0.895 for PSDs expressed as probability density functions and measured at different dilutions confirm that the apparent overestimation is a statistical artifact. In sum, serial dilution does not affect the measured PSD as long as every bin contains a significant number of counts.

To find the optimal dilution of biofluids to perform MRPS, three compounding factors require consideration. First, the concentration of the sample must be within the specified range of the cartridge, which is difficult to estimate without prior knowledge. The cartridges that the nCS1 uses are prone to clog during measurements when sample concentrations approach the upper bound of the manufacturer specified range. However, the nCS1 prevents frequent pore clogging with embedded pre-filters in the cartridges and mitigates pore clogging with a *Clear Constriction* feature of the acquisition software [18, 20].

Second, if the only objective of the MRPS measurement is to determine total particle concentration, then it is sufficient to detect 1,000 particles to realize a Poisson error of 3.2%. Conversely, if the objective of the MRPS measurement is to measure a broad PSD, the profile of the PSD and the required statistical significance per bin dictate the minimum number of particle detection events that are necessary to ensure low Poisson error. The third consideration that dictates the optimal dilution is the length of time spent on each measurement. From Fig 6B, diluting plasma 1,000-fold detected an average of 46 particles per 100 s, while the 10-fold dilution resulted in > 10,000 particles per 100 s. Yet, diluting plasma 10-fold increases (i) the probability of pore clogging, (ii) the noise floor, and (iii) the likelihood of swarm detection. The time to collect data is a fraction of the total time spent on the instrument, which involves the time it takes to prime the microfluidics (150–300 s), run the instrument buffer between each 10 s acquisition (10–20 s), and perform data analysis/processing (> 600 s). Thus, the total time it takes to complete a measurement can vary substantially and is ultimately driven by the objective of the MRPS measurement (report a concentration vs. measure a complete PSD). It is our opinion after performing this analysis, that the cartridges perform at peak capacity when the biofluid is diluted near the middle of the concentration range specified by the manufacturer for each cartridge (1 × 10^9^ mL^−1^ for TS-300 or TS-400). This ensures the highest achievable counting efficiency with the lowest likelihood of clogging, resulting in the shortest possible measurement time.

To investigate the agreement in measured concentration between cartridges with overlapping LoD, we measured the PSDs of plasma, PC3 CM, and urine. TS-400 and TS-2000 cartridges quantified the PSDs of plasma and PC3 CM from 75 nm up to 2 μm, while TS-300, TS-400, and TS-2000 cartridges quantified the PSD of urine from 65 nm up to 2 μm. From Fig 7, applying a power-law function to the measured concentrations resulted in a strong agreement for plasma (R^2^ = 0.958), PC3 CM (R^2^ = 0.936), and urine (R^2^ = 0.946), which leads to two key findings: (i) overlapping two or three different cartridges with different LoD generates reproducible results over a broad dynamic range of size (65 or 75 nm up to 2 μm) and concentration (1 × 10^4^—1 × 10^12^ mL^−1^), and (ii) validation of the assumption that the particles in plasma, PC3 CM, and urine follow a power-law function below the LoD with a high degree of agreement between the measured concentrations and modeled fits.

## Conclusion

We developed a reliable and reproducible operating procedure to measure the PSD and concentration of EVs together with other particles in biofluids with MRPS (Table 2). This study did not assess the accuracy of MRPS or the effect of sample purification on the resulting PSD. Purified samples, in theory, could reduce the background noise during MRPS measurements, as purification techniques reduce the background noise in both similar and orthogonal particle size characterization techniques (TRPS, NTA, FCM, cryo-EM) [15, 23, 34–38] and could presumably decrease the LoD. We now have a reliable and reproducible operating procedure for measuring the PSD and concentration of EVs together with other particles in biofluids, which is necessary before evaluating accuracy and precision. The manufacturer’s operation manual recommends spiking samples with calibration beads of known diameter prior to performing a measurement and scaling the reported PSD by the measured diameter of the calibration beads. However, we recommend caution when following the manufacturer’s recommendation as spiking biological fluids with calibration beads may result in the development of a “protein corona” due to the rapid adsorption of proteins in solution to the surface of the nanoparticle, which increases the diameter of a calibration bead by as much as 30 nm [39–41]. Future work will elucidate the accuracy and precision of MRPS using reference particles with known size distribution.

## Supporting information

S1 File The supplementary materials document provides the MIFlowCyt-EV documentation. (PDF)

S2 File The supplementary materials document provides insight into the manufacturer’s background subtraction technique. (PDF)

S3 File The supplementary materials document provides insight into the differences between MRPS and TRPS. (PDF)
