# Supplementary material for: Standardized procedure to measure the size distribution of extracellular vesicles together with other particles in biofluids with microfluidic resistive pulse sensing
Source: PLoS One. 2021 Apr 1;16(4):e0249603. doi: 10.1371/journal.pone.0249603 (PMC8016234; doi:10.1371/journal.pone.0249603)
Supplement: S1 File — (PDF) [file pone.0249603.s001.pdf]

MIFlowCyt-EV of “Standardized procedure to measure the size distribution of extracellular vesicles together with other particles in biofluids with microfluidic resistive pulse sensing”

Michael Cimorelli[1,2,3*], Rienk Nieuwland[2,3], Zoltán Varga[5], Edwin van der Pol[2,3,4]

1 Department of Chemical & Biological Engineering, Drexel University, Philadelphia, United States of America
2 Department of Clinical Chemistry, University of Amsterdam location AMC, Amsterdam, the Netherlands
3 Vesicle Observation Center, University of Amsterdam location AMC, Amsterdam, the Netherlands
4 Department of Biomedical Engineering and Physics, University of Amsterdam location AMC, Amsterdam, the Netherlands
5 Biological Nanochemistry Research Group, Institute of Materials and Environmental Chemistry, Research Center for Natural Sciences, Budapest, Hungary

* mjc428@drexel.edu

Flow cytometry

Experimental design

The aim of flow cytometry (A60-Micro, Apogee Flow Systems, Hemel Hempstead, UK) experiments were to determine if the concentration of extracellular vesicles (EVs) in plasma or urine were affected by five different diluents: (1) 0.01% Tween 20 (v/v), (2) 1% Tween 20 (v/v), (3) 0.1% BSA (w/v), (4) 0.1% Triton X-100 (v/v) (negative control), and (5) DPBS (positive control). We hypothesized that the concentration of EVs in plasma or urine diluted in 0.1% BSA (w/v) would have a similar concentration to EVs in plasma or urine diluted in DPBS, while the concentration of EVs in plasma or urine diluted in 0.01% Tween 20 (v/v), 1% Tween 20 (v/v), 0.1% Triton X-100 (v/v) would have a significantly lower concentration. As a positive control, we measured the concentration of EVs in plasma and urine in DPBS. As a negative control, we measured the concentration of EVs in plasma and urine in 0.1% Triton X-100 (v/v), a strong non-ionic detergent that is known to lyse EVs [1].

All samples were measured using an autosampler, which facilitates subsequent measurements of samples in a 96-well plate. The entire study involved three 96-well plates that were measured on separate days. Two of the three studies had well plates that contained a buffer-only control, while all studies contained well plates that had antibody in buffer controls and isotope controls that correspond to the labels in the well plate. Scatter calibrations were performed daily, while fluorescence and flow rate calibrations were performed once. To automatically determine optimal sample dilutions, apply calibrations, determine and apply gates, generate reports with scatter plots and generate data summaries, we developed and applied custom-build software (MATLAB R2018b, Mathworks, Natick, MA, USA).

Sample dilutions

To prevent swarm detection [2], we diluted all samples in DPBS to realize a count rate $\leq 5.0 \times 10^3$ events per second, as motivated by earlier work [3].

EV staining

EVs in platelet free plasma (PFP) were double labeled with CD61-APC and lactadherin-FITC, while EVs in urine were single labeled with lactadherin-FITC. Prior to staining, antibodies were diluted into DPBS and centrifuged at 18,890 g for 5 min to remove aggregates. Table 1 details the reagents and antibody concentrations used during staining. To stain, 20 μL of pre-diluted PFP or pooled urine was incubated with 2.5 μL of antibodies or isotype controls and kept in the dark for 2 hours at room temperature. The staining reaction was stopped by adding 200 μL of DPBS.

Table 1. Overview of staining reagents. Characteristics being measured, analyte, analyte detector, reporter, isotype, clone, concentration, manufacturer, catalog number and lot number of used staining reagents. The antibody concentration during measurements was 11.1-fold lower than the antibody concentration during staining.

Characteristic measured	*Analyte*	*Analyte detector*	*Reporter*	*Isotype*	*Clone*	*Concentration during staining ($\mu g\ mL^{-1}$)*	*Manufacturer*	*Lot number*
Integrin	Human CD61	Anti-human CD61antibody	APC	IgG1	VI-PL2	50	eBioscience	2026494
Glyco-protein	Lactadherin	Lactadherin	FITC	n.a.	n.a.	83	Haematologic Technologies	JJ0307

APC: allophycocyanin; FITC: fluorescein isothiocyanate; IgG: immunoglobulin G

Buffer-only control

Each 96-well plate had at least 1 well with clean DPBS, which was measured with the same flow cytometer and acquisition settings as all other samples. The mean count rate for DPBS, 0.01% Tween 20 (v/v), 1.0% Tween 20 (v/v), 0.1% Triton X-100 (v/v), and 0.1% BSA (w/v) was 18.9 events per second, 77.2 events per second, 186.3 events per second, 135.3 events per second, and 185.8 events per second, respectively.

Buffer with reagents control

Each 96-wellplate contained a buffer with reagent control for each reagent (Table 1), which was measured with the same flow cytometer and acquisition settings as all other samples. For particles with a diameter > 200 nm and a refractive index < 1.42, as reported in this study, we measured an average of 1.0, 0.0, 3.0, 1.0, and 7.0 APC+ events per second for CD61-APC in DPBS, 0.01% Tween 20 (v/v), 1.0% Tween 20 (v/v), 0.1% Triton X-100 (v/v), and 0.1% BSA (w/v), respectively. For particles with a diameter > 200 nm and a refractive index < 1.42, as reported in this study, we measured an average of 2.0, 26.0, 1.00, 2.0, and 2.0 FITC+ events per second for lactadherin-FITC in DPBS, 0.01% Tween 20 (v/v), 1.0% Tween 20 (v/v), 0.1% Triton X-100 (v/v), and 0.1% BSA (w/v), respectively.

Unstained controls

We have not performed unstained controls.

Isotype controls

Table 1 shows an overview of the used isotype controls. For particles with a diameter > 200 nm and a refractive index < 1.42, as reported in this study, we measured on average of 0.0 APC+ events per second for IgG-APC in DPBS, 1 APC+ events per second for IgG-APC in 0.1% BSA (w/v), 2.0 APC+ events per second for IgG-APC in 0.01% Tween 20 (v/v), 2.0 APC+ events per second for IgG-APC in 1% Tween 20 (v/v), 0.0 APC+ events per second for IgG-APC in 0.1% Triton X-100 (v/v).

Trigger channel and threshold

Based on the buffer-only control (49.8 events s^{-1}), the acquisition software was set up to trigger at 14 arbitrary units SSC, which is equivalent to a side scattering cross section of 10 nm^2 (Rosetta Calibration, v1.11, Exometry, Amsterdam, The Netherlands).

Flow rate quantification

Each measurement day, we used 110-nm FITC beads with a specified concentration (Apogee calibration beads, Apogee Flow Systems, Hemel Hempstead, UK) to calibrate the flow rate of the A60-Micro. The adjusted flow rate is 3.01 μL/min and the measured median flow rate is 2.85 ± 0.14 uL/min (mean ± standard deviation). Because the A60-Micro is equipped with a syringe pump with volumetric control, we assumed a flow rate of 3.01 μL/min for all measurements. Figure 1 shows the measured flow rate of the A60-Micro on the day the experiments were performed.

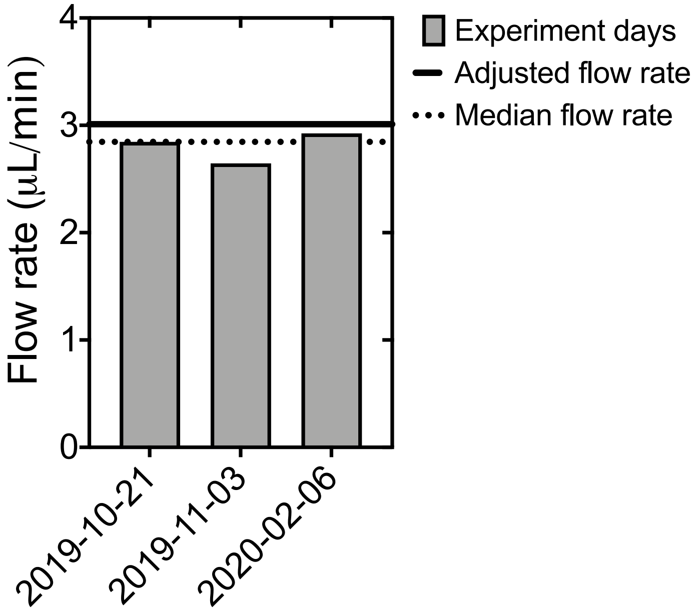

Fig 1. Measured flow rate of the A60-Micro on the day experiments were performed. The adjusted flow rate is 3.01 µL/min and the median flow rate is 2.85 µL/min. The measured flow rate was within 5.46% of the adjusted flow rate.

Fluorescence calibration

Calibration of the fluorescence detectors from arbitrary units (a.u.) to molecules of equivalent soluble fluorochrome (MESF) was accomplished using 2 μm Q-APC beads

(2321-175, BD, Franklin Lakes, NJ, USA) and QuantiBright FITC-5 MESF beads (13734, Bangs Laboratories, Inc., Fishers, IN, USA). Figure S2A and S2B illustrate the relationship between the measured MFI and specified MESF for APC (A) and FITC (B). For each measurement, we added fluorescent intensities in MESF to the flow cytometry data files by custom-build software (MATLAB R2018a) using following equation:

$$I(MESF) = 10^{(a \times log_{10} I(a.u.)+b)} \tag{1}$$

where I is the fluorescence intensity, and a and b are the slope and the intercept of the linear fits in Figure 2, respectively.

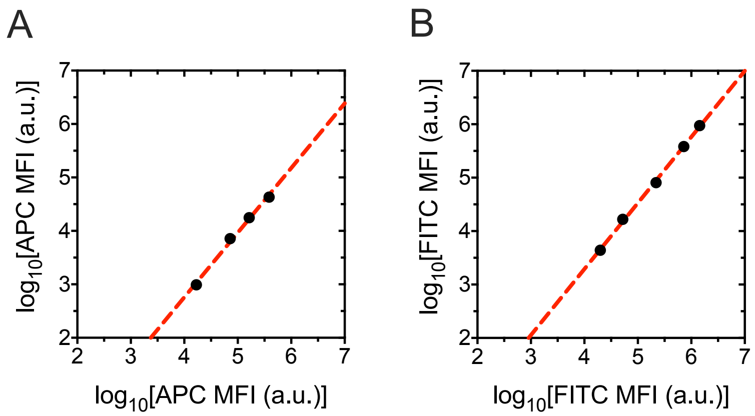

Fig 2. Calibration of the fluorescence detectors from arbitrary units (a.u.) to molecules of equivalent soluble fluorochrome (MESF). Logarithmic MESF versus logarithmic mean fluorescence intensity (MFI) for APC (A) and FITC (B). Data (symbols) are fitted with a linear regression, resulting in a slope of 1.21 and intercept of -2.08 for APC and a slope of 1.24 and intercept of -1.66 for FITC.

EV diameter and refractive index approximation

Flow-SR was applied to determine the size and refractive index of particles and to improve specificity by enabling label-free differentiation between EVs and lipoprotein particles [4, 5]. Flow-SR was performed as previously described [4, 5]. Lookup tables were calculated for diameters ranging from 10 to 1,000 nm, with step sizes of 1 nm, and refractive indices from 1.35 to 1.80 with step sizes of 0.001. The diameter and refractive index of each particle were added to the .fcs file by custom-build software (MATLAB R2018a). Because Flow-SR requires accurate measurements of both FSC and SSC, we applied Flow-SR only to particles with diameters > 200 nm and fulfilling the condition:

$$SSC(nm^2) > -0.7 \times FSC(nm^2) + 3 \tag{2}$$

MIFlowCyt checklist

The MIFlowCyt checklist is added to Table 2.

Table 2. Overview of staining reagents. Characteristics being measured, analyte, analyte detector, reporter, isotype, clone, concentration, manufacturer, catalog number and lot number of used staining reagents. The antibody concentration during measurements was 11.1-fold lower than the antibody concentration during staining

Requirement	Please Include Requested Information
1.1. Purpose	To determine if the concentration of extracellular vesicles (EVs) in pooled plasma or urine were affected by five different diluents: (1) 0.01% Tween 20 (v/v), (2) 1% Tween 20 (v/v), (3) 0.1% BSA (w/v), (4 100 (v/v) (negative control), and (5) DPBS (positive control).
1.2. Keywords	Tween 20, Triton X-100, extracellular vesicles, BSA, Lysis
1.3. Experiment variables	Buffer
1.4. Organization name and address	Amsterdam University Medical Centers Location Academic Medical Centre Meibergdreef 9 1105 AZ Amsterdam, The Netherlands
1.5. Primary contact name and email address	Michael Cimorelli, mjc428@drexel.edu
1.6. Date or time period of experiment	October 2019, November 2019, and February 2020
1.7. Conclusions	Concentration of EVs in pooled plasma was not affected by DPBS or 0.1% BSA (w/v). Concentration of EVs in pooled plasma decreased significantly 0.1% Triton X-100 (v/v) and 0.01% or 1% Tween 20 (v/v). This same result was observed in pooled urine, except 0.01% Tween 20 (v/v) resulted in a mild increase in EV concentration.
1.8. Quality control measures	All samples were measured using an autosampler, which facilitates subsequent measurements of samples in a 96-well plate. Each well plate contained buffer-only controls (Buffer-only control), antibody in buffer controls (Buffer with reagents control), and isotype controls (Isotype controls). The flow rate was cross calibrated with Rosetta Calibration (Exometry, Amsterdam, The Netherlands). Fluorescence detectors were calibrated (Fluorescence calibration with 2 μm Q-APC beads (2321-175, BD, Franklin Lakes, NJ) and QuantiBright FITC-5 MESF beads (13734, Bangs Laboratories, Inc., Fishers, IN). FSC and SSC were calibrated with Rosetta Calibration (v1.11, EV diameter and refractive index approximation).
1.9 Other relevant experiment information	The entire study involved three 96-well plates that were measured within several months.
2.1.1.1. Sample description	Thawed platelet free plasma and thawed pooled urine

2.1.1.2. Biological sample source description	Whole blood was collected using a 21G needle in 3-mL citrate Vacutainers (BD Biosciences, San Jose, CA) from 20 healthy participants (10 males, 10 females) with informed consent. To remove cells and isolate plasma, blood was centrifuged at 2,500 g for 15 minutes at room temperature using a Rotina 380R centrifuge (Hettich, Tuttlingen, Germany). The plasma (10 mm above the buffy coat) was transferred to new tubes and centrifuged once more at 2,500 g for 15 minutes at room temperature to remove residual platelets. Subsequently, the plasma was pooled, and 100-μL aliquots were snap frozen in liquid nitrogen and stored at -80 °C until use. Urine was obtained from fasted healthy participants (5 males) with informed consent. The urine was pooled in 8 aliquots of 50 mL and centrifuged twice in 50-mL Greiner tubes, 180 g for 10 minutes at 4 °C and 1,560 g for 20 minutes at 4 °C, to remove cells using a Rotina 380R centrifuge (Hettich, Tuttlingen, Germany). The pooled urine was separated into 1-mL aliquots and snap frozen in liquid nitrogen and stored at -80 °C until use. Before analysis, pooled plasma was thawed for 1 minute in 37 °C water while pooled urine was thawed for 3 minutes in 37 °C water to dissolve amorphous salts. All samples were stored on ice until the start of an experiment.
2.1.1.3. Biological sample source organism description	Whole blood – 20 healthy participants (10 male, 10 female)<br>Urine – 5 fasted healthy participants (5 males)
2.2 Sample characteristics	Platelet free plasma (PFP) is expected to contain EVs, lipoproteins and proteins.<br>Pooled urine is expected to contain primarily EVs.
2.3. Sample treatment description	Please see EV staining.
2.4. Fluorescence reagent(s) description	Please see Table 1.
3.1. Instrument manufacturer	Apogee, Hemel Hempstead, UK
3.2. Instrument model	A60-Micro
3.3. Instrument configuration and settings	Samples were analysed for 2 minutes at a flow rate of 3.01 µL/min on an A60-Micro, equipped with a 405-nm laser (100 mW), 488-nm laser (100 mW) and 638-nm laser (75 mW). The trigger threshold was set at SSC 14 arbitrary units, corresponding to a side scattering cross section of 10 nm^2 (Rosetta Calibration). For FSC and SSC, the PMT voltages were 380 V and 360 V, respectively. For all detectors, the peak height was analyzed. APC signals were collected with the 638-D Red (peak) detector (long pass 652-nm filter, PMT voltage 510 V). FITC signals were collected with the 488-Green (peak) detector (525/50 nm band pass filter, PMT voltage 520 V).
4.1. List-mode data files	A summary of all flow cytometry scatter plots and gates applied are available via `https://www.doi.org/10.6084/m9.figshare.12622007.v2`
4.2. Compensation description	No compensation was required because no fluorophore combinations were used that have overlapping emission spectra.
4.3. Data transformation details	No data transforms were applied.

4.4.1. Gate description	To automatically apply gates, generate pdf reports with scatter plots, and summarize the data in a table, custom-build software (MATLAB R2018b) was used. Please find below a description of the gates.<br>First, only events that were collected during time intervals, for which the count rate was within 25% of the median count rate, were included.<br>Second, residual platelets were excluded by applying a gate at the side scattering cross section (< 2,000 nm^2) and, depending on the fluorescence label, at a fluorescence channel. For samples stained with CD61-APC, CD61-APC aggregates were omitted by selecting data fulfilling the condition stated by:<br>$SSC(nm^2) > APC(MSEF) + 3$.<br>Third, to include particles within the dynamic range of Flow-SR [3], particles with a diameter > 200 nm and fulfilling the condition of equation 2 were included.<br>Fourth, to exclude false positively labeled chylomicrons and thus primarily include EVs, only particles with a refractive index < 1.42 were included.<br>Fifth, fluorescence gates were automatically determined with an algorithm (MATLAB R2018b) and applied. Lower bounds of the fluorescent gates for plasma were 150 MESF for CD61-APC and 350 MESF for lactadherin-FITC, while for urine were 600 MESF for lactadherin-FITC.
4.4.2. Gate statistics	The number of positive events was corrected for flow rate, measurement time, and dilutions performed during sample preparation.
4.4.3. Gate boundaries	On overview of all gates can be found in the compressed data summary files `https://www.doi.org/10.6084/m9.figshare.12622007.v2`

EV number concentration

The concentrations reported in the manuscript describe the number of particles (1) that exceeded the SSC threshold, corresponding to a side scattering cross section of 10 nm^2, (2) that were collected during time intervals, for which the count rate was within 25% of the median count rate, (3) with a diameter > 200 nm as determined by Flow-SR [4], (4) fulfilling the condition of equation 2, (5) having a refractive index < 1.42 to omit false positively labeled chylomicrons, and (6) are positive at the corresponding fluorescence detector(s), per mL of plasma or urine.

Data sharing

A summary of all flow cytometry scatter plots and gates applied are available via `https://www.doi.org/10.6084/m9.figshare.12622007.v2`

References

1. Osteikoetxea X, Sódar B, Németh A, Szabó-Taylor K, Pálóczi K, Vukman KV, et al. Differential detergent sensitivity of extracellular vesicle subpopulations. Organic and Biomolecular Chemistry. 2015;13(38):9775–9782. doi:10.1039/c5ob01451d.

2. Van der pol E, Van Gemert MJC, Sturk A, Nieuwland R, Van Leeuwen TG. Single vs. swarm detection of microparticles and exosomes by flow cytometry. Journal of Thrombosis and Haemostasis. 2012;10(5):919–930. doi:10.1111/j.1538-7836.2012.04683.x.
3. van der Pol E, Buntsma N. EDTA stabilizes the release of platelet-derived EVs. 2019;doi:https://doi.org/10.6084/m9.figshare.c.4753676.v3.
4. van der Pol E, de Rond L, Coumans FAW, Gool EL, Böing AN, Sturk A, et al. Absolute sizing and label-free identification of extracellular vesicles by flow cytometry. Nanomedicine: Nanotechnology, Biology, and Medicine. 2018;14(3):801–810. doi:10.1016/j.nano.2017.12.012.
5. de Rond L, Libregts SFWM, Rikkert LG, Hau CM, van der Pol E, Nieuwland R, et al. Refractive index to evaluate staining specificity of extracellular vesicles by flow cytometry. Journal of Extracellular Vesicles. 2019;8(1). doi:10.1080/20013078.2019.1643671.
