# Supplementary material for: Standardized procedure to measure the size distribution of extracellular vesicles together with other particles in biofluids with microfluidic resistive pulse sensing
Source: PLoS One. 2021 Apr 1;16(4):e0249603. doi: 10.1371/journal.pone.0249603 (PMC8016234; doi:10.1371/journal.pone.0249603)
Supplement: S2 File — (PDF) [file pone.0249603.s002.pdf]

Supplemental Materials for "Standardized procedure to measure the size distribution of extracellular vesicles together with other particles in biofluids with microfluidic resistive pulse sensing"

Michael Cimorelli[1,2,3*], Rienk Nieuwland[2,3], Zoltán Varga[5], Edwin van der Pol[2,3,4]

1 Department of Chemical & Biological Engineering, Drexel University, Philadelphia, United States of America
2 Department of Clinical Chemistry, University of Amsterdam location AMC, Amsterdam, the Netherlands
3 Vesicle Observation Center, University of Amsterdam location AMC, Amsterdam, the Netherlands
4 Department of Biomedical Engineering and Physics, University of Amsterdam location AMC, Amsterdam, the Netherlands
5 Biological Nanochemistry Research Group, Institute of Materials and Environmental Chemistry, Research Center for Natural Sciences, Budapest, Hungary

* mjc428@drexel.edu

Background on the removal of electronic noise

The presence of electrical noise means that particle detection events near the LoD (limits of detection) have larger relative contributions from electrical noise than do those further from the LoD. This results in particle detection events near the limit of detection being more difficult to distinguish from electrical noise despite strict filtering criteria (transit time, symmetry, diameter, and signal to noise ratio). To overcome this limitation, the contribution of false positives – electrical noise that is included as a particle detection event – is estimated using a background subtraction. The raw voltage signal shown in Fig 1B and 1C is composed of broadband electrical noise and real particle events that are superimposed on a flat baseline. The electrical noise signal is constant and symmetrical about the baseline, while real particle events cause peaks in the measured voltage on only one side of the baseline, as shown in Fig 1B. In the background subtraction method, the peak detection algorithm is applied to both the particle (positive) and non-particle (negative) sides of the baseline. On the particle side, the resulting particle size distribution (PSD) is composed of both real particle events and electrical noise induced false positives, while on the non-particle side the resulting PSD is only composed of electrical noise induced false positives. Because the electrical noise is symmetric about the baseline, this gives rise to a direct measurement of the contribution of electrical noise on the particle side. Thus, the background subtraction removes this electrical noise distribution from the real PSD to eliminate any contribution of electrical noise in the measurement.
