# Supplementary material for: Standardized procedure to measure the size distribution of extracellular vesicles together with other particles in biofluids with microfluidic resistive pulse sensing
Source: PLoS One. 2021 Apr 1;16(4):e0249603. doi: 10.1371/journal.pone.0249603 (PMC8016234; doi:10.1371/journal.pone.0249603)
Supplement: S3 File — (PDF) [file pone.0249603.s003.pdf]

Supplemental Materials for "Standardized procedure to measure the size distribution of extracellular vesicles together with other particles in biofluids with microfluidic resistive pulse sensing"

Michael Cimorelli[1,2,3*], Rienk Nieuwland[2,3], Zoltán Varga[5], Edwin van der Pol[2,3,4]

1 Department of Chemical & Biological Engineering, Drexel University, Philadelphia, United States of America
2 Department of Clinical Chemistry, University of Amsterdam location AMC, Amsterdam, the Netherlands
3 Vesicle Observation Center, University of Amsterdam location AMC, Amsterdam, the Netherlands
4 Department of Biomedical Engineering and Physics, University of Amsterdam location AMC, Amsterdam, the Netherlands
5 Biological Nanochemistry Research Group, Institute of Materials and Environmental Chemistry, Research Center for Natural Sciences, Budapest, Hungary

* mjc428@drexel.edu

Differences between microfluidic resistive pulse sensing and tunable resistive pulse sensing

Both microfluidic resistive pulse sensing (MRPS) and tunable resistive pulse sensing (TRPS) are based on the Coulter Principle. However, this is an oversimplification of the differences between the technologies. The fundamental similarity between the techniques is that they both use changes in electrical properties (voltage, current, resistance) to infer information about particle size and concentration, yet, both technologies accomplish this differently.

TRPS uses a standard two-electrode system (identical to the original Coulter Counter) to cause sample flow through the nanopore, while MRPS employs a three-electrode system where the outer two electrodes serve as a voltage-divider [1–4] This voltage-divider limits the influence of both electrophoresis or electroosmosis of sample through the system, while both of these phenomena are important variables at small pore size during TRPS measurements [5, 6]. MRPS uses a pneumatic system to control and direct flow of sample throughout the instrument, while TRPS uses a variable pressure module (to control static head pressure) and primarily relies on an applied electric field to direct flow of sample through the system [1, 2, 4]. MRPS enables the ability to embed pre-filters upstream of the nanoconstriction to prevent large structures from clogging the nanoconstriction (see *DOI: 0.1038/nnano.2011.24* Figure 1a or 2a), while TRPS does not enable the inclusion of pre-filters [1–3]. As mentioned in the manuscript, MRPS cartridges have rigid, pre-calibrated pores of polydimethylsiloxane, while TRPS uses sensing pores of polyurethane that can be stretched to tune the pore size [1, 2, 4, 7]. Calibration of any resistive pulse amplitude to particle size depends linearly on the volume of the pore [3–5, 7]. Therefore, the fixed pore design of MRPS cartridges allows for pre-calibration of cartridges that are manufactured in the same lot, while the elastic nature of the TRPS system

fundamentally prevents this. As a result of the variable pore geometry, TRPS measurements require a calibration using polystyrene standards of a known volume prior to every measurement. MRPS directly measures the flow rate of particles through the nanoconstriction using transit time, while TRPS does not measure sample flow rate and therefore cannot estimate the volume of sample analyzed. MRPS measures the total sample volume and divides the number of particles counted by the volume measured to determine concentration. TRPS determines particle concentration via calibration standards without direct measurement of the sample flow rate [1, 2]. MRPS is able to measure particle concentrations from 50 nm – 10 µm with a concentration bandwidth of 1×10^4 - 5×10^{11} mL^{-1} (link) while TRPS can measure particles from 40 nm – 10 µm with a concentration bandwidth of 1×10^5 - 1×10^{11} mL^{-1} (link).

References

1. Izon Science Ltd. Izon qNano - Operation Manual. 2014;K(January):1–42.
2. Spectradyne LLC. Spectradyne nCS1 - Operation Manual. 2017;2.3.6:1–35.
3. Fraikin JL, Teesalu T, McKenney CM, Ruoslahti E, Cleland AN. A high-throughput label-free nanoparticle analyser. Nature Nanotechnology. 2011;6(5):308–313. doi:10.1038/nnano.2011.24.
4. Vogel R, Coumans FAW, Maltesen RG, Böing AN, Bonnington KE, Broekman ML, et al. A standardized method to determine the concentration of extracellular vesicles using tunable resistive pulse sensing. Journal of Extracellular Vesicles. 2016;5(1). doi:10.3402/jev.v5.31242.
5. Weatherall E, Willmott GR. Applications of tunable resistive pulse sensing. Analyst. 2015;140(10):3318–3334. doi:10.1039/c4an02270j.
6. Willmott GR, Fisk MG, Eldridge J. Magnetic microbead transport during resistive pulse sensing. Biomicrofluidics. 2013;7(6):1–17. doi:10.1063/1.4833075.
7. Coumans FAW, van der Pol E, Böing AN, Hajji N, Sturk G, van Leeuwen TG, et al. Reproducible extracellular vesicle size and concentration determination with tunable resistive pulse sensing. Journal of Extracellular Vesicles. 2014;3(1):1–8. doi:10.3402/jev.v3.25922.
